# Outcomes of Sphincter of Oddi Manometry When Performed in Low Volumes

**DOI:** 10.1155/2011/435806

**Published:** 2011-06-02

**Authors:** John P. Rice, Bret J. Spier, Deepak V. Gopal, Anurag Soni, Mark Reichelderfer, Patrick R. Pfau

**Affiliations:** Section of Gastroenterology and Hepatology, Department of Medicine, University of Wisconsin Hospital and Clinics, H6/516 Clinical Science Center, Madison, WI 53792-5124, USA

## Abstract

*Background*. Sphincter of Oddi manometry is a highly specialized procedure associated with an increased risk of procedural complications. Published studies have typically been performed in large volume manometry centers. *Objective*. To examine the outcomes and complication rate of SOM when performed in small volumes. *Design*. Retrospective analysis at a tertiary care referral hospital that infrequently performs Sphincter of Oddi manometry. Patient records were reviewed for procedural details, patient outcomes, and complications after sphincter of Oddi manometry. *Results*. 36 patients, 23 (23 type II sphincter of Oddi dysfunction (SOD), 13 type III SOD) underwent sphincter of Oddi manometry and were followed up for mean of 16 months. Nine Type II patients (90%) with elevated basal sphincter pressures noted symptom improvement after sphincterotomy compared with only 3 patients (43%) of the patients with normal basal pressures. In type III SOD, 7 patients had elevated basal SO pressure and underwent sphincterotomy. Three patients (43%) improved. There were six (16%) procedure-related complications. There were four cases of post ERCP pancreatitis (11%), all of which were mild. *Conclusion*. In low numbers, sphincter of Oddi manometry can be performed successfully and safely by experienced biliary endoscopists with results that are comparable to large volume centers.

## 1. Introduction

Sphincter of Oddi dysfunction (SOD) is a term used to describe epigastric or right upper quadrant pain syndromes attributed to dyskinesia or stenosis of the sphincter of Oddi. It is also commonly termed postcholecystectomy syndrome. Data has suggested that in patients with sphincter of Oddi stenosis, endoscopic sphincterotomy may be of symptomatic benefit [1, 2]. However, identifying this patient population remains a challenge for physicians, both clinically and in research endeavors. Part of this challenge is the realization that SOD is likely a heterogenous group of disorders, influenced by other factors including psychological stressors, and overlaps with functional GI disorders including irritable bowel syndrome [3, 4]. Presently, patients with suspected SOD are stratified into three different categories based on objective laboratory and radiologic findings coupled with pain [1, 5, 6]. 

Endoscopic retrograde cholangiopancreatography (ERCP) with sphincter of Oddi manometry (SOM) is considered the “gold standard” in the diagnosis of SOD. Sphincter hypertension, defined as basal sphincter pressures above 40 mmHg, is considered manometric evidence of SOD [7]. Current standard of practice is to perform endoscopic sphincterotomy without manometry in patients classified as type I SOD. These patients are felt to have papillary stenosis and will benefit from sphincterotomy without further investigation [8]. In patients categorized as type II SOD, documentation of abnormal biliary sphincter pressures with SOM is advised before proceeding to sphincterotomy. This recommendation is based largely on two randomized clinical trials [1, 2]. Type III SOD is an even more challenging clinical situation as these patients lack objective evidence of sphincter dysfunction and tend to have overlap with underlying functional disorders, such as irritable bowel syndrome [3, 4]. Current recommendations suggest that complete noninvasive diagnostic testing and empiric trials with antispasmotics or antidepressants be attempted prior to consideration of SOM [5, 6]. Despite the above research, controversy remains regarding SOD and the use of SOM as a diagnostic tool. 

ERCP with SOM is considered to have a significant risk of postprocedural complication. The most highly publicized and feared complication of SOM is post-ERCP pancreatitis. Research has shown this risk to be higher than the general population, with risk ranging from as low as 3% all the way up to greater than 20% [9, 10]. In addition to risks specific to SOM, other standard risks of ERCP also are problematic, including post-sphincterotomy hemorrhage, infection, and perforation [11].

To date, the vast majority of research regarding SOM has occurred at high-volume SOM centers with specific expertise and interest in the performance and interpretation of SOM. Therefore, the applicability and reproducibility of published data outside of such institutions where SOM is frequently performed and studied is unclear. Further given the risk of SOM and complexity, it has been suggested that its performance be limited to those performing sphincter manometry in high numbers. The purpose of this study was to examine patient outcomes and postprocedural complication risk of SOM when performed infrequently in low volumes. 

## 2. Methods

### 2.1. Study Design and Patient Selection

Patients underwent ERCP with SOM from January 2003 to July 2009. The patient's electronic medical records were reviewed to determine prior radiology and laboratory data, medical therapy (i.e., tricyclic antidepressants (TCA), selective serotonin reuptake inhibitors (SSRI), antispasmotics (i.e., dicyclomine), opioids) before and after ERCP, technical success of the procedure, manometric findings, whether sphincterotomy was performed, presence and severity of postprocedure complications, improvement of symptoms, and duration of followup. The University of Wisconsin IRB approved this protocol. 

### 2.2. Procedure Details

All patients underwent ERCP using a Pentax side-viewing duodenoscope (Model: ED 3490 TK and ED 3270 K, Pentax of America Inc., Montvale, NJ). ERCP and manometry was performed by a single, experienced biliary endoscopist that performs in excess of 400 ERCPs per year. Twenty-nine patients underwent SOM with general anesthesia. Seven patients underwent SOM under conscious sedation. 

 Once the duodenoscope was in place opposite the major papilla, SOM was performed using the following protocol. A manometry catheter (Cook Scientific SOM 18-L-Lehman-NG Manometry Catheter, Wilson-Cook Medical, Winson-Salem, NC) was inserted into the bile duct, measuring intraductal pressures. Basal sphincter pressures were then measured with standard station pull-through technique. Three pull-throughs were performed with sustained basal sphincter pressure measurements obtained from both the proximal and distal sensors. Mean basal sphincter pressure was calculated from the basal sphincter pressures obtained during each pull-through. If mean basal sphincter pressure was greater than 40 mmHg, sphincterotomy was then performed. If mean basal sphincter pressure was less than 40 mmHg, empiric sphincterotomy was performed under the discretion of the performing endoscopist based on patient symptoms, duration of symptoms, and severity of symptoms. Pancreatic duct stenting was done at the endoscopist's discretion. Generally if the pancreatic duct was not injected and the bile duct was cannulated in 1–3 attempts a pancreatic stent was not placed. 

### 2.3. Variables Collected

Prior to SOM, laboratory and radiology results were reviewed including previous liver function tests, abdominal ultrasonographic procedures, CT scans, MRIs, or MRCPs. Patients were then classified as sphincter of Oddi type I, II, or III based on Rome III revised Milwaukee Group Classification [5, 6]. For biliary SOD, patients were defined as Type I SOD if they noted biliary-type pain associated with an elevated alkaline phosphatase, aminotransferase, or total bilirubin greater than two times the upper limit of normal (ULN) and evidence of a dilated common bile duct (CBD) greater than 10 mm on noninvasive imaging and were not included in this study. Patients were classified as Type II SOD if they had pain with only laboratory abnormalities or imaging abnormalities. They were classified as Type III SOD if they had pain but no laboratory or imaging abnormalities.

At the time of ERCP with SOM, the variables collected were successful cannulation rate, ability to perform manometric measures, the mean basal sphincter pressure recorded by manometry, whether or not sphincterotomy was performed, and whether a pancreatic duct stent was placed. 

Postprocedure, the variables collected were postprocedural complications and their severity as assessed by consensus criteria [11]. The postprocedural complications that were examined were post ERCP pancreatitis, perforation, post sphincterotomy bleeding, cholangitis, and admission to the hospital post-procedure. Complications were identified either immediately after procedure, at a 24 hour post procedure call back, or at 30 days through a chart review. 

 Finally, patient outcomes were assessed based on symptomatic improvement expressed by the patient in clinic followup. The patient symptom improvement was categorized as no response, partial response, or complete response based on patient reporting. Symptoms were categorized based on their symptoms at last known followup. This was done primarily to account for any placebo effect seen after performance of sphincterotomy. 

### 2.4. Statistical Analysis

The primary outcome analyzed was improvement in patient symptoms with endoscopic sphincterotomy. For the purpose of this analysis, patients were grouped according to sphincter of Oddi type and then by presence or absence of sphincter hypertension. Fisher's exact test was then used to compare the proportion of patients with symptom relief after sphincterotomy. 

### 2.5. Exclusion Criteria

Patients were excluded from the analysis if they were classified Type I SOD given current recommendations for these patients to undergo sphincterotomy given the high concordance with SOD. Patients were also excluded if they had no followup or inadequate followup to document response to treatment/sphincterotomy. 

## 3. Results

### 3.1. Patient Characteristics

**Figure 1:**
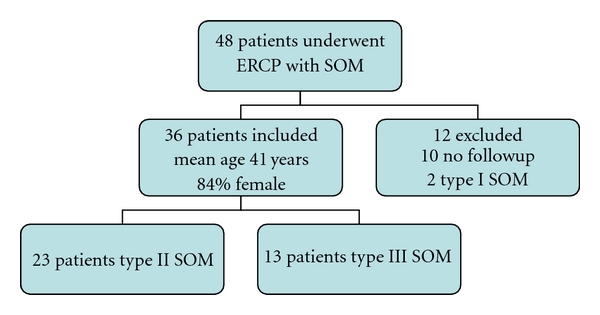
Patient inclusion and demographics by sphincter of Oddi dysfunction type.

Forty-eight patients underwent SOM during the study period (mean of 7.38 cases per year). Thirty-six patients were included in the final analysis because they had complete follow-up data (Figure 1). Thirty-one (84%) were female with a mean of 41 years of age. Twenty-three patients (64%) were classified as type II SOD, while 13 patients (36%) were classified as type III SOD. Cannulation rate was 100% with the ability to carry out sphincter of Oddi manometry in all patients studied. After SOM, patients were followed for a mean of 16 months (range of 1–45 months). 

### 3.2. Manometry Results and Patient Outcome

#### 3.2.1. SOD type II

Of the 23 patients classified as type II SOD, 10 had a mean basal sphincter pressure of greater than 40 mmHg during SOM (Table 1). All ten of these patients underwent endoscopic sphincterotomy at the time of ERCP. Nine (90%) patients experienced sustained subjective improvement in symptoms with sphincterotomy at time of last followup. Three patients had complete resolution of their pain, 6 had some degree of improvement in pain, and 1 patient experienced no relief in symptoms. 

Of the remaining 13 SOD type II patients that had basal sphincter pressures less than 40 mmHg, 7 (54%) had a sphincterotomy performed. In contrast to type II SOD patients with elevated basal sphincter pressures, only 3 (43%) with sphincter pressures less than 40 who underwent a sphincterotomy experienced any improvement in symptoms in followup (90% versus 43%, *P* = .10). One patient in this group experienced complete symptoms resolution (30% versus 14%, *P* > .50). In comparison, two (33%) of the 6 patients with pressures less than 40 mmHg in which sphincterotomy was not performed experienced symptom improvement during the follow-up period. This patient population was managed medically with therapy aimed at treating functional abdominal pain. 

#### 3.2.2. SOD type III

Thirteen patients had been classified as SOD type III (Table 1). Seven subsequently were found to have elevated basal SO pressures and all underwent endoscopic sphincterotomy. Of these 7 patients, 3 (43%) had symptomatic improvement after sphincterotomy. Six patients did not have manometric evidence of elevated basal sphincter pressures. However, two patients did undergo sphincterotomy and one patient did report an improvement in symptoms (*P value *>.50). Among the remaining four patients who did not undergo sphincterotomy, none experienced an improvement in symptoms during followup. 

### 3.3. Medical Therapy

Prior to SOM, 26 (72.2%) patients were on some form of medical therapy. Of the 26 patients, 18 (78.3%) patients had type II SOD and 8 (61.5%) patients had type III. Postprocedure, 29 patients were on medical therapy at last followup. All 10 patients that did not undergo sphincterotomy were on medical therapy. Of the 26 patients that underwent sphincterotomy, seven patients (6 type II SOD, 1 type III SOD) got off of medication for functional pain altogether. Nine patients remained on medical therapy but experienced improvement in symptoms after sphincterotomy (6 type II SOD, 3 type III SOD). The remaining 10 patients (5 type II SOD, 5 type III SOD) did not benefit from sphincterotomy and were all on medical therapy at the end of study. 

### 3.4. Complications

Six patients (16%) had postprocedural complications. Four patients (11%) had post-ERCP pancreatitis. All four cases were considered mild by consensus criteria published by Cotton et al., in 2009 [11]. All four patients were discharged from the hospital within 48 hours of admission and recovered without sequelae. One patient (2.8%) had a moderate postsphincterotomy hemorrhage. She was hospitalized and required transfusion of three units of packed red blood cells. One patient (2.8%) developed moderate cholangitis post-procedure. During her initial procedure, she was found to have normal basal sphincter pressures and no intervention was performed. However, over the next 24 hours she became febrile to 38.6 degrees Celsius. Her serum total bilirubin rose 5.9 mg/dL. She underwent repeat ERCP, and a sphincterotomy was performed. She recovered without sequelae. Of note, three patients did have pancreatic duct stents placed at the time of ERCP in an attempt to avoid post-ERCP pancreatitis. One of these patients was one of the four patients that developed pancreatitis. Finally, nine patients (25%) were admitted to the hospital for postprocedural abdominal pain. None of these patients had objective evidence of pancreatitis, cholangitis, or perforation. Seven of these patients were type II SOD and 2 were type III SOD. Seven had undergone biliary sphincterotomy during the procedure. SOD type and performance of sphincterotomy were not associated with probability of postprocedural pain. All nine patients were discharged within 48 hours. 

## 4. Discussion 

The diagnosis and management of patients with sphincter of Oddi dysfunction remains a challenge and a controversy for gastroenterologists. Principle to that challenge is determining which patient population may benefit from endosocopic sphincterotomy. In addition to classification systems based on objective data, randomized studies have suggested that SOM is beneficial in the identification of true sphincter hypertension and thus potential benefit from sphincterotomy [1, 2]. Studying sphincterotomy versus sham procedure, Geenen et al. showed a 91% of patients classified as type II SOD with sphincter hypertension benefitted symptomatically from sphincterotomy [1]. Toouli et al. showed a benefit of sphincterotomy only in patients with sphincter hypertension [2]. Despite the promising data in these randomized studies, skepticism remains regarding the accuracy and reproducibility of these data. Several nonrandomized studies have called into question the utility of SOM in identifying patients that benefit from sphincterotomy and in the need for manometry in all suspected Type II and III patients [12, 13]. In addition, research regarding SOM primarily occurs in centers with extensive experience and interest in the performance and interpretation of SOM. For example, in a recently published paper from a single center, 5352 patients had ERCP with SOM over a 13-year period, averaging over 400 manometry cases per year [14]. Similarly, in a recent paper from Cotton et al., over a 12-year period performed over 1300 biliary SOM were performed at one institution, corresponding to over 100 SOM cases per year [11]. It is unknown if ERCP with SOM can and should be performed outside of such large SOM centers. 

In this study, we examined the outcomes and safety of SOM in the diagnosis and treatment of SOD at a center where SOM is performed far less frequently. In general, about 7 patients in our institution undergo SOM annually. Despite these small numbers, our data is similar to previously published data. In our study, those patients classified as type II SOD with manometric evidence of sphincter hypertension seemed to benefit from sphincterotomy (Table 1). Additionally, patients with type II SOD without sphincter hypertension and patients with type III SOD demonstrated less demonstrable benefit from sphincterotomy. This data is in agreement with published guidelines that sphincter of Oddi manometry is best performed in type II and Type III SOD with sphincterotomy reserved for those with documented sphincter hypertension [1, 6]. We also found that even when rarely performed SOM can be used to predict who will have a benefit from endoscopic sphincterotomy. 

The overall complication rate of 16% in this study fell well within expected values with no major complications. Acute pancreatitis, the most feared complication of SOM, occured in only 11% of patients and all of the cases were mild being hospitalized for less than 5 days with no sequelae. This value also falls within previously published rates [9, 10]. It is important to note however that while our center is a low-volume SOM center, it is a tertiary referral ERCP center. All of the ERCPs in this study were performed by an experienced biliary endoscopist that performs in excess of 400 ERCPs per year. Previous studies have documented that low ERCP volume is associated with an increased risk of postprocedural complications [15]. Therefore, it should be reinforced that although abundant experience in the performance of SOM may not be a necessary prerequisite for SOM safety, whether SOM effectiveness and safety can be reproduced in any center particularly those with overall low volume ERCPs cannot be definitively stated. 

There are several weaknesses to this study. First is the nature of the study with a small patient population. We performed this study with the purpose to see if SOM could be performed successfully in small numbers but this made true statistical comparison difficult. While the data trended toward statistical significance, the total population was small and the data did not reach significance. In addition, no patient followup was available for twelve patients. The main reason for lack of patient followup is that these patients often were from geographic areas that were significantly far from our center and were referred for SOM. Finally, outcomes were based on patient perception of symptom improvement. Scoring systems to assess symptom and quality of life improvement were not used. In addition, as with all functional intestinal disorders, the impact of placebo effect cannot be underestimated.

In conclusion, SOM is perhaps the most technically challenging ERCP procedure in a challenging patient population. Most research on SOM has been performed in large volume centers with highly experienced gastroenterologists in the performance and interpretation of SOM. There has been little research published on SOM in a “real world” scenario outside of larger research studies and SOM centers. This study reflects that SOM can be performed safely with acceptable risk in centers that perform SOM infrequently. In addition, it did reproduce previously published data suggesting that manometry studied patients with sphincter hypertension are the most likely population to benefit from sphincterotomy, and SOM when done in low numbers can also be effective in the treatment of suspected SOD patients. 

##  Funding and Conflict of Interests

None of the authors have received funding for this study or have conflicts of interest to disclose. 

## Figures and Tables

**Table 1:** Clinical outcomes of patients stratified by SOD type, manometric findings, and whether sphincterotomy was performed.

SOD type	Mean basal SO pressure	*N*	Symptom improvement (%)	*P* value
SOD type II	>40 mmHg (total)	10	90	
	Sphincterotomy	10	90	
	No sphincterotomy	0	n/a	.10
	<40 mmHg (total)	13	38	
	Sphincterotomy	7	43	
	No sphincterotomy	6	33	

SOD type III	>40 mm Hg (total)	7	43	
	Sphincterotomy	7	43	
	No sphincterotomy	0	n/a	>.50
	<40 mm Hg (total)	6	17	
	Sphincterotomy	2	50	
	No sphincterotomy	4	0	
